# Fault Diagnosis of Rolling Bearing Based on HPSO Algorithm Optimized CNN-LSTM Neural Network

**DOI:** 10.3390/s23146508

**Published:** 2023-07-19

**Authors:** He Tian, Huaicong Fan, Mingwen Feng, Ranran Cao, Dong Li

**Affiliations:** 1 National Demonstration Center for Experimental Mechanical and Electrical Engineering Education, Tianjin University of Technology, Tianjin 300384, China; tianhe@tjut.edu.cn (H.T.);; 2 Tianjin Key Laboratory for Advanced Mechatronic System Design and Intelligent Control, School of Mechanical Engineering, Tianjin University of Technology, Tianjin 300384, China; 3 School of Electrical Engineering and Automation, Tianjin University of Technology, Tianjin 300384, China; 4 Tianjin Key Laboratory for Control Theory & Applications in Complicated Industry Systems, Tianjin 300000, China

**Keywords:** intelligent fault diagnosis, convolutional neural network, long short-term memory, hybrid particle swarm optimization, rolling bearing

## Abstract

The quality of rolling bearings is vital for the working state and rotation accuracy of the shaft. Timely and accurately acquiring bearing status and early fault diagnosis can effectively prevent losses, making it highly practical. To improve the accuracy of bearing fault diagnosis, this paper proposes a CNN-LSTM bearing fault diagnosis model optimized by hybrid particle swarm optimization (HPSO). The HPSO algorithm has a strong global optimization ability and can effectively solve nonlinear and multivariate optimization problems. It is used to optimize and match the parameters of the CNN-LSTM model and dynamically find the optimal value of the parameters. This model overcomes the problem that the parameters of the CNN-LSTM model depend on empirical settings and cannot be adjusted dynamically. This model is used for bearing fault diagnosis, and the accuracy rate of fault diagnosis classification reaches 99.2%. Compared with the traditional CNN, LSTM, and CNN-LSTM models, the accuracy rates are increased by 6.6%, 9.2%, and 5%, respectively. At the same time, comparing the models with different optimization parameters shows that the model proposed in this paper has the highest accuracy. The experimental results verified the superiority of the HPSO algorithm to optimize model parameters and the feasibility and accuracy of the HPSO-CNN-LSTM model for bearing fault diagnosis.

## 1. Introduction

In recent years, rolling bearings, as the essential parts supporting the rotation of shafts, have played an important role in mechanical equipment, so fault diagnosis technology for rolling bearings has attracted much research attention [1,2,3]. At present, rolling bearing fault diagnosis technology is mainly divided into methods based on signal processing and methods based on artificial intelligence [4,5,6,7]. Signal processing-based methods usually involve preprocessing bearing vibration signals and extracting time- and frequency-domain features using signal processing techniques. The artificial intelligence-based method adaptively extracts fault features in vibration signals by learning historical experience data. Compared with signal processing-based methods, artificial intelligence-based methods overcome time-consuming and subjective problems and show more significant potential and advantages.

At present, artificial intelligence-based fault diagnosis techniques include BP neural networks, convolutional neural networks (CNNs) and long short-term memory (LSTM) neural networks [8,9,10]. CNNs can effectively identify signals for feature extraction and fault identification and can also be used as a classifier with local correlation [11,12,13]. LSTM has serial correlation, which can solve the problem of continuous input and long-term sequence [14,15,16]. However, CNNs lack memory and cannot extract dynamic features in data, while LSTM has a limited effect when handling high-dimensional data. However, LSTM must face the challenge of long-term dependence when processing sample sequences that are too long, and it is difficult to identify faults of similar features [17,18,19]. Therefore, CNNs and LSTM neural networks are combined to form a CNN-LSTM model, which uses the CNN layer to extract the short-term feature information of the fault adaptively and as the input to the LSTM layer after dimensionality reduction. The LSTM layer further learns the fault feature information and trains the neural network model to classify faults finally.

The developed artificial neural network model was used to evaluate the wear of different ball-bearing materials. The test results show that the results obtained by the proposed model are almost similar to the experimental results, which proves the correctness of the neural network model in bearing testing applications [20]. Based on the mixed model of a CNN and a single classifier to diagnose bearing faults, comparison analysis with traditional fault feature extraction methods shows that the CNN has a good feature extraction ability [21], but the CNN network cannot extract dynamic features, and it is difficult to reflect the correlation and importance between features. The combination method of long-short-term memory and large-margin nearest neighbour (LSTM-LMNN) uses the initialization technology of orthogonal weight in LSTM to memorize key fault information during the parameter update process to complete fault diagnosis. LSTM realizes the memory function in time, preventing the gradient from disappearing [22]. However, LSTM is prone to uneven distribution of feature information, and it is difficult to identify similar features. An end-to-end solution based on 1D is a CNN-LSTM model that extracts sensor vibration signals’ spatial and temporal features and then connects them to diagnose better bearing faults [23]. Combining 1D CNN and LSTM into a unified structure to identify bearing faults [24]. A fault diagnosis method based on a deformable CNN, DLSTM and transfer learning strategy. The deformable CNN enhances the ability to extract features using fixed geometric structures and further encodes the sequential information in the CNN through DLSTM. Then, the transfer learning strategy is implemented to identify and classify bearing faults [25]. However, the parameters of the CNN-LSTM model are difficult to determine and cannot be adjusted with training. Different parameters correspond to different structures, and the diagnostic results vary greatly. Dividing the time series into multiple channels, using the CNN-LSTM method, and applying an attention-based mechanism to improve the performance are then used to predict bearing remaining life [26]. Optimize the number of neurons in the CNN-LSTM model with the GA algorithm and then conduct comparative experiments with CNN-LSTM and other models. The results showed that the model has better performance [27]. However, the GA algorithm calculation is large, the number of iterations required is high, and the general parameter optimization time will be longer. Using the PSO-optimized CNN-LSTM network model, PSO repeatedly searches and optimizes the complex hyperparameter space of the CNN-LSTM model, extracts features through CNN analysis, and LSTM models’ temporal information to accurately differentiate and identify user permissions [28]. However, the PSO algorithm easily falls into a local optimum, and the optimized parameters may not be optimal.

In summary, the CNN-LSTM model is widely used in bearing fault diagnosis. However, the parameters of the CNN-LSTM network model are difficult to set, usually need to be pre-set based on experience, and cannot be dynamically adjusted during the training process. Different parameter settings correspond to different network structures, resulting in large differences in diagnostic results. For this reason, the HPSO algorithm has a better global optimization ability than the traditional PSO algorithm and GA. This study uses the HPSO algorithm to optimize the parameters of the CNN-LSTM model, the optimal number of convolution kernels, the number of hidden layer nodes, the learning rate and the maximum number of iterations. This method realizes the dynamic adjustment of the model parameters and the search for the optimal value. Based on this, an HPSO-CNN-LSTM bearing fault diagnosis model is proposed in this paper, and the optimal parameters are applied to the CNN-LSTM model and used for bearing fault diagnosis. The experimental results show that, compared with traditional deep learning networks, the model has better performance in both classification accuracy and diagnosis accuracy and exhibits superior fault diagnosis capabilities.

The remaining sections of this paper are as follows. Section 2 briefly introduces the CNN, LSTM and HPSO algorithms. Section 3 proposes an HPSO-CNN-LSTM model. Section 4 describes the setup of the experimental platform and the collection and selection of fault data. The model is trained and tested on the fault data, and the accuracy of fault classification and the fault diagnosis ability of different models are obtained. Section 5 summarizes the full text.

## 2. Traditional Network Model Algorithm

### 2.1. Convolutional Neural Networks

A convolutional neural network (CNN) is a deep feedforward neural network generally composed of a convolutional, pooling, and fully connected layer [29,30,31]. The convolution layer performs feature extraction and mapping in the local perception field of view using the convolution kernel on the input feature map. The convolution kernel moves on the feature map and performs the convolution operation with the data in the sensory field of view. The mathematical expression of the convolution operation [32] is as follows:

(1) $$x_{i}^{l}=f(W_{i}^{l}*X^{\left(l-1\right)}+b_{i}^{l})$$

In the formula, $x_{i}^{l}$ represents the i feature of the output value of the lth floor; $W_{i}^{l}$ indicates the weight matrix of the ith convolution nucleus on the lth floor; ∗ operations represent the convolutional calculation; $X^{\left(l-1\right)}$ is the output of layer l−1th; $b_{i}^{l}$ represents the bias item; and the f function represents the activation function of the output. After the convolution operation, there is usually an excitation layer, in which the excitation layer needs to use a nonlinear activation function to perform nonlinear mapping on the output of the convolution layer to increase the fitting ability of the model. Using a rectified linear unit (*ReLU*) as the activation function of the convolutional layer, its mathematical formula is as follows:

(2) ReLU=0, x<0x, x≥0

The pooling layer reduces the dimension of the feature map after the convolution layer, performs feature selection, and reduces the number of features to alleviate the overfitting phenomenon. Pooling sampling methods are generally divided into maximum pooling sampling and average pooling sampling. Using maximum pooling sampling, the expression is as follows:

(3) $$y_{i}^{\left(i+1\right)}(j)=\max x_{i}^{j}(k),\;k\in D_{j}$$

In the formula, $y_{i}^{\left(i+1\right)}(j)$ is the element in the feature map of the ith feature map of the l+1th layer after pooling; $D_{j}$ is the jth pooling area; and $x_{i}^{j}(k)$ means that the ith feature map of the lth layer is within the scope of the pooling kernel.

The fully connected layer is a traditional multilayer perceptron. Its neurons are all connected to the neurons of the previous layer. It mainly refits the features, integrates the differentiated local information between different categories, and reduces the loss of feature information. The output layer then uses a softmax activation function to combine the previously extracted features for probability distribution and classification [33,34]. The expression is as follows:

(4) $$p(y_{j})=\frac{\exp(y_{j})}{\sum_{k=1}^{m}\exp(y_{k})}$$

In the formula, $p(y_{j})$ is the probability output of the neurons passing through the softmax activation function; $\exp(y_{j})$ is the output value of the jth neurons in the output layer; and m is the number of classifications of the target.

### 2.2. LSTM

**Figure 1:**
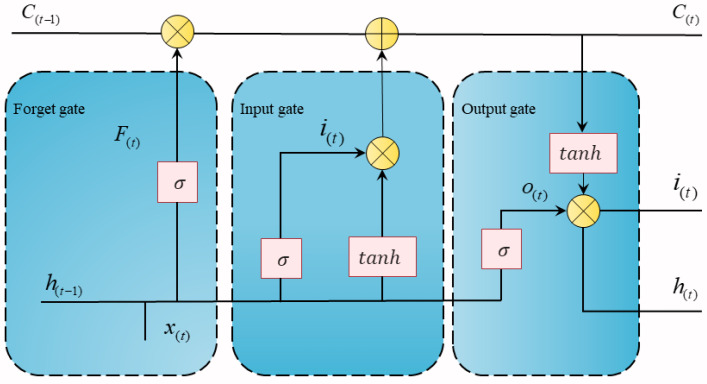
LSTM structure.

Long short-term memory neural networks (LSTM, long short-term memory) are fully connected neural network structures with self-loop feedback. Compared with the traditional recurrent neural network (RNN), LSTM neural networks have a more complex architecture. LSTM introduces three special “gate” structures in the hidden layer: a forget gate, an input gate and an output gate. These gates are selective and can screen and regulate information. In addition, LSTM also introduces a cell state, which is used to represent the information at the current moment and passed to the subsequent LSTM layer at the next moment [35,36]. These characteristics endow the LSTM network with the ability to effectively solve the long-distance dependence problem and the gradient disappearance problem to learn the long-term and short-term correlation information of the time series and effectively transmit and express the information in the long-term series [37,38]. Figure 1 shows the basic structure of the LSTM network.

At current time t, the input data of the time sequence are expressed as $x_{t}$, the cell status is $C_{t}$, and the output is $h_{t}$. The values of the three doors in LSTM [39] are as follows.

(1) Forget gate $f_{t}$: LSTM will dynamically adjust according to the new input and the output of the previous time and selectively remember or forget the historical information to control the influence of historical information on the neuron information at the current time.

(5) $$f_{t}=\sigma (\omega_{f}h_{t-1}+\omega_{f}x_{t}+b_{f})$$

In the formula, σ represents the sigmoid activation function; ω is the weight matrix of the gate; b is the bias term of the gate; and $h_{t-1}$ is the output of the neuron at the previous time.

(2) Input gate $i_{t}$: The selection of new input information controls the effect of the current information on the neuronal information and acts as flow control.

(6) $$i_{t}=\sigma (\omega_{i}h_{t-1}+\omega_{i}x_{t}+b_{i})$$

(3) Unit status value $C_{t}$:

(7) $$C_{t}=f_{t}*C_{t-1}+i_{t}*\tanh(\omega_{c}h_{t-1}+\omega_{c}x_{t}+b_{c})$$

In the formula, tanh  represents the hyperbolic tangent activation function.

(4) Output gate $y_{t}$: The selection of the output under the current time controls the output information to the neuron information and the output of the unit state.

(8) $$y_{t}=h_{t}=\sigma (\omega_{o}h_{t-1}+\omega_{o}x_{t}+b_{o})*\tanh C_{t}$$

In the formula, $y_{t}$ is the current neuronal output.

### 2.3. Hybrid Particle Swarm Optimization Algorithm (HPSO)

Particle swarm optimization (PSO) is an intelligent stochastic optimization algorithm with simple operation and fast convergence speed. However, as the number of iterations increases, the similarity between particles increases, and it is easy to fall into a locally optimal solution. The genetic algorithm (GA) has good global optimization ability, but the convergence speed is slow, and the convergence accuracy is low. Therefore, this study adopts hybrid particle swarm optimization (HPSO) and introduces the crossover and mutation operations in the GA into the PSO algorithm. The crossover operation makes the particles cross each other, reflecting the idea of information exchange in the algorithm, and the mutation operation can not only maintain the diversity of the population but also avoid the occurrence of premature convergence. Compared with a single algorithm, the HPSO algorithm has better global optimization ability, thus improving the performance of the algorithm.

The particle position and velocity crossover operations [40] are expressed as follows:

(9) $$\left\{{}_{x_{j}^{\left(k+1\right)}=(1-\alpha_{1})\cdot x_{i}^{k}+\alpha_{1}x_{j}^{k}}^{x_{i}^{\left(k+1\right)}=\alpha_{1}x_{i}^{k}+(1-\alpha_{1})\cdot x_{j}^{k}}\right.$$

(10) $$\left\{{}_{v_{j}^{\left(k+1\right)}=(1-\alpha_{2})\cdot v_{i}^{k}+\alpha_{2}v_{j}^{k}}^{v_{i}^{\left(k+1\right)}=\alpha_{2}v_{i}^{k}+(1-\alpha_{2})\cdot v_{j}^{k}}\right.$$

In the formula, $\alpha_{1}$ and $\alpha_{2}$ are random numbers in the interval [0, 1]; the particles $x_{i}^{k+1}$, $x_{j}^{k+1}$, $v_{i}^{k+1}$, and $v_{j}^{k+1}$ are the children of $x_{i}^{k}$, $x_{j}^{k}$, $v_{i}^{k}$, and $v_{j}^{k}$ after crossover, respectively.

The quality of the solution depends heavily on the variation rate, and a Gaussian variation operation is applied to the current individual extreme value $p_{best}$ using random perturbation. For the current optimal extreme value of each particle, the variation operation is applied with a certain probability, and β obeys the $gauss\left(0,1\right)$ distribution with the following formula:

(11) $$p_{best}^{d}=p_{best}^{d}(1+0.5\beta )$$

**Figure 2:**
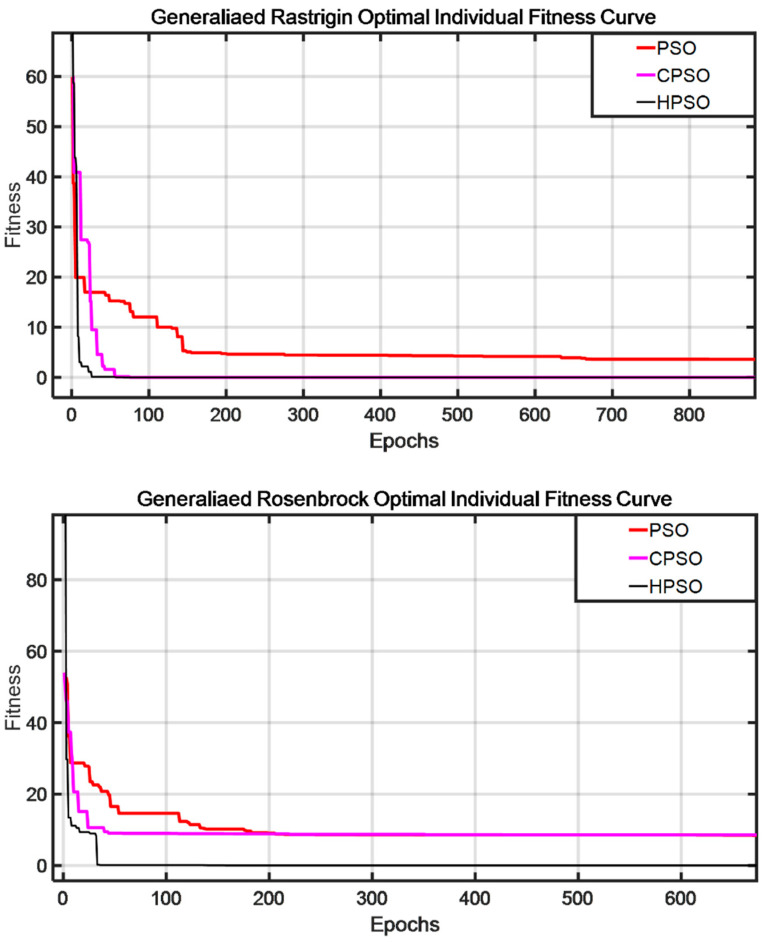
PSO, CPSO and HPSO optimal individual fitness curves.

The multimodal function generalized Rastrigin and ill-conditioned function generalized Rosenbrock are used to evaluate the basic particle swarm optimization (PSO), chaotic particle swarm optimization (CPSO) and particle swarm optimization based on the GA (HPSO). Figure 2 shows three different optimal individual fitness curves of the algorithms. The HPSO algorithm’s fitness curve is significantly better than the other two. Its convergence speed is faster, the optimal fitness value is reached at approximately the 10th iteration, and the curve is smoother. Therefore, compared with the standard particle swarm optimization algorithm and the chaotic particle swarm optimization algorithm, the HPSO algorithm has the advantages of simple operation and easy implementation, but it improves the performance of each step of the GA and PSO and introduces crossover and mutation in the GA in the PSO algorithm. Therefore, it has evident progress in population diversity and convergence speed. It can effectively solve nonlinear and multivariable optimization problems, does not easily fall into local optima, and has excellent global stable optimization ability.

## 3. HPSO-CNN-LSTM Models

In bearing fault diagnosis, the CNN-LSTM model uses a CNN to adaptively extract short-term feature information and reduce the dimension as the LSTM input and uses LSTM to learn fault feature information and train the neural network model. Combining the two models can complement each other and improve fault diagnosis performance. However, the parameters of the CNN-LSTM model are usually determined theoretically by experience, and the model performance largely depends on selecting appropriate optimal parameters. Choosing an optimal set of parameters is labour-intensive and time-consuming, and the parameters cannot be automatically adjusted as the model changes. The current common parameter adjustment methods include the network search strategy and random search strategy. However, when the deep learning neural network has many hyperparameters, the efficiency of the network search is low. Although the random search method has high efficiency, its stability is poor, and it is easy to ignore some key error messages. Therefore, in response to this problem, we need an efficient and stable parameter tuning optimization method to improve the performance of the CNN-LSTM model in bearing fault diagnosis.

The HPSO algorithm is a parameter optimization algorithm with better performance. After the particles pass the crossover and mutation operations of the GA, the particle diversity is better, and it is not easy to fall into the local optimum. Different particles move towards the individual optimal position, the global optimal position is moved, and the particle information of the optimal path is found according to the information propagation of each other to find the global optimal solution. In the CNN-LSTM model, there are multiple hyperparameters. Among them, the number of convolution kernels, the number of hidden layer nodes, the learning rate and the maximum number of iterations have a great impact on the performance of the network model. Selections that are too large or too small will lead to network performance degradation. This paper selects these four most critical parameters for optimization, uses the HPSO algorithm to adjust and find the optimal solution dynamically, determines a set of optimal network model parameters, and then constructs the optimized HPSO-CNN-LSTM model to carry out bearing troubleshooting. The CNN part consists of two convolutional layers and two maximum pooling layers, using the ReLU function as the activation function. The LSTM part is modelled using a stack of two LSTM networks.

**Figure 3:**
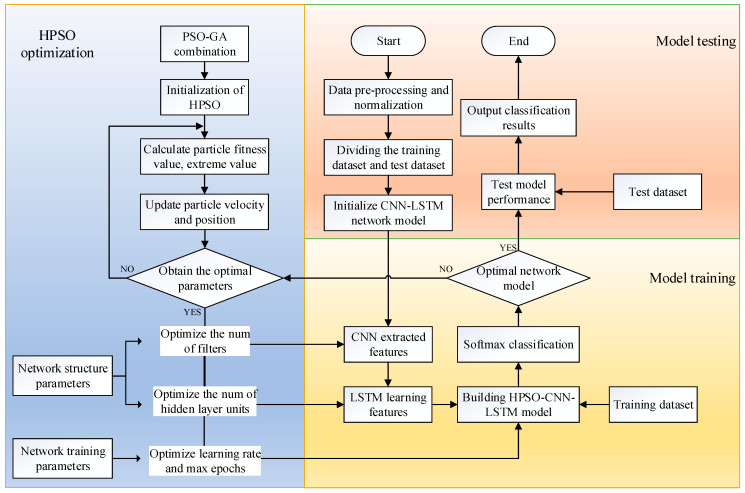
Fault diagnosis flow chart of the HPSO-CNN-LSTM model.

Figure 3 is a flow chart of the HPSO-CNN-LSTM network model, and the main steps are as follows:

Step 1: Preprocess the data, divide the experimental data into a training set, verification set and test set, and normalize the data;

Step 2: Initialize the parameters and structure of the CNN-LSTM network model. Select the number of convolution kernels in the model, the number of hidden layer nodes, the learning rate and the maximum number of iterations as the parameter objects to be optimized;

Step 3: Initialize the hybrid particle swarm optimization algorithm and calculate the particle fitness, individual, and group extreme values. Use the mean square error (MSE) as the evaluation standard to determine the particle fitness value;

Step 4: Update the velocity and position of the particles and record the results for each iteration. The Adam algorithm is used for error backpropagation. When the iteration is completed, or the optimal parameter is found, the termination condition is met, the optimal parameter value is obtained, and the model parameters are updated layer by layer. If not satisfied, return to step 3 and iterate again;

Step 5: Use the CNN layer to extract the features of the bearing fault data. After the maximum pooling layer pooling operation, reduce the data dimension, and input the feature data after dimension reduction to the LSTM layer for fault feature learning;

Step 6: Construct the HPSO-CNN-LSTM model based on the optimal hyperparameters and classify the output through the softmax layer;

Step 7: The optimal network model is obtained through cyclic training of the training set, and the test set is input to the HPSO-CNN-LSTM network model to test the model’s performance. Finally, the fault diagnosis and classification results are output.

## 4. Experience

### 4.1. Experimental Setup and Data Acquisition

**Figure 4:**
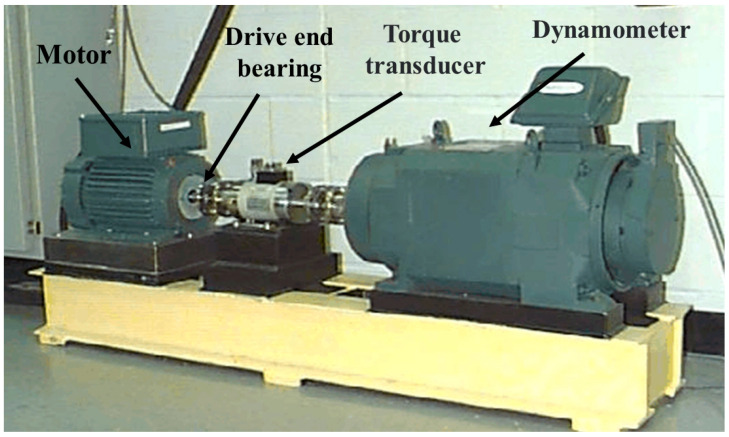
Case Western Reserve University bearing experimental data acquisition equipment.

The experimental data come from the Case Western Reserve University (CWRU) Bearing Data Center, and the basic layout of the experimental test rig is shown in Figure 4. It consists of a 2-horsepower Reliance Electric motor driving a shaft on which a torque transducer and encoder are mounted. Torque is applied to the shaft via a dynamometer and electronic control system. In experimental testing, faults with diameters of 0.007, 0.014 and 0.028 inches were implanted in drive end bearings, bearing type 6205-2RS JEM SKF. The motor is machined using electrical discharge machining (EDM). The faults were seeded on the rolling elements, inner and outer races, and each failed bearing was (individually) remounted on the test rig. Then a 1 hp motor load was run at a constant speed (approximate motor speed of 1772 rpm). In each test, the acceleration in the vertical direction is measured on the drive end-bearing housing (DE) [41].

This experiment selects the data of the drive end bearing, the bearing model is 6205-2 RS JEM SKF, the motor speed is 1772 rpm, the load is 1hp, and the sampling frequency is 12 kHz. The types of bearing faults are divided into four categories according to the location and size of the bearing fault: normal state, inner ring fault, outer ring fault and rolling element fault. Each type of fault is divided into 0.007 inches, 0.014 inches and 0.028 inches according to the size, and the bearing status is divided into ten types in total.

In this experiment, the acceleration data of the driving end of the bearing are selected as the model input. First, data expansion and classification processing are performed on the acceleration data of the driving end. According to different types of faults, the acceleration data of the driving end are divided into ten sample sets, and the data are overlapped and sampled to expand the characteristic value of the fault and improve the accuracy of fault classification. The ten sample sets correspond to ten different categories of bearing diagnosis status as the model classification output, which is normal, inner ring fault, outer ring fault and rolling element fault when the diameter of the drive end bearing fault is 0.007 inches and the drive end bearing fault diameter inner race failure, outer race failure and rolling element failure at 0.014 inches, drive end bearing failure inner race failure, and outer race failure and rolling element failure at 0.028-inch diameter. After data preprocessing, each dataset contains 300 sets of data, and each set of data has 4096 feature values. In model training, we select the first 250 data groups from each dataset as training samples and determine the optimal network model through data identification and classification training. The remaining 50 sets of data are used as test samples and input into the trained network model to test the model’s performance, calculate the model evaluation index, and output the fault diagnosis and classification results, error values and classification diagrams. These results are used to evaluate the accuracy and error of the network model.

### 4.2. Simulation Result Analysis

MATLAB is used to simulate and analyse the HPSO-CNN-LSTM fault diagnosis model, in which the network model selects the number of convolution kernels, the number of hidden layer nodes, the learning rate and the maximum number of iterations as the optimization object for algorithm optimization. To better select the combination of hyperparameters, we first set the hyperparameter search space and the value ranges of the optimized hyperparameters. Specifically, the number of convolution kernels is [5, 50], the range of the number of hidden layer nodes is [20, 200], the range of the learning rate is [0.01, 0.2], and the range of the maximum number of iterations is [10, 100]. In the HPSO algorithm, the particle population size is 10, the evolution times are 10, the crossover probability is 0.5, and the mutation probability is 0.5.

The parameters of the CNN-LSTM network model are optimized through HPSO, and the parameters of the optimized model are obtained. The model parameter settings are shown in Table 1.

**Figure 5:**
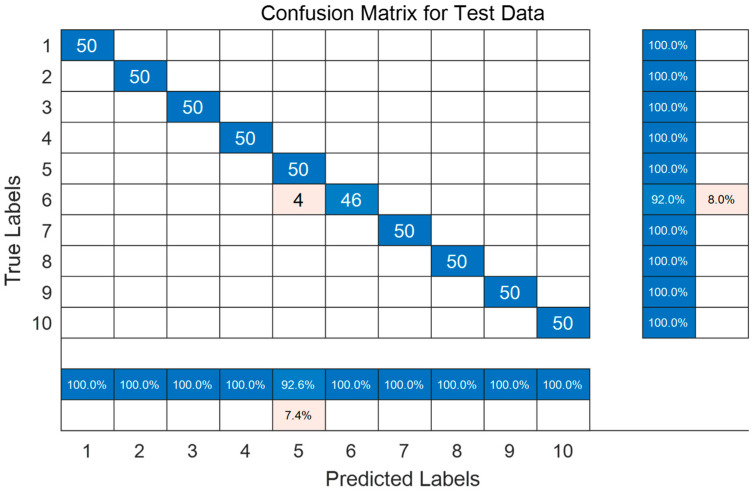
Confusion matrix for the test data.

**Figure 6:**
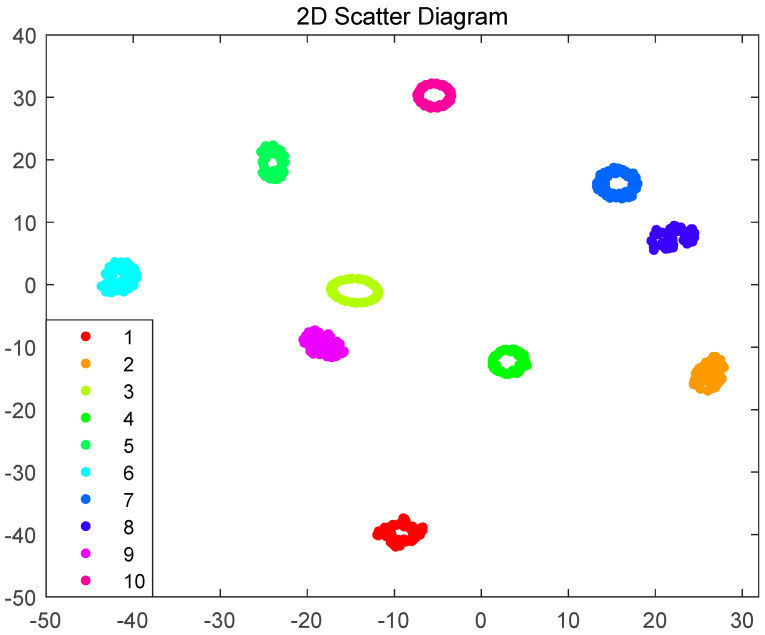
2D scatter diagram.

The four optimized parameters are the number of filters = [6, 6], number of hidden layer units = [197, 197], learning rate = 0.0649, and max epochs = 76. Using the HPSO-CNN-LSTM network model to test the bearing fault test set data, it can be concluded that the accuracy of fault diagnosis and classification reaches 99.2%. Figure 5 shows the confusion matrix, where the abscissa represents the predicted fault label, the ordinate represents the actual fault label, and the elements on the diagonal indicate that the predicted result is consistent with the actual result label. Categories 1–10 represent normal, inner race failure, outer race failure and rolling element failure at 0.007” diameter of drive end bearing failure, inner race failure, outer race failure and rolling element failure at 0.014-inch diameter of drive end bearing failure faults, and drive end bearing faults, inner race faults, outer race faults and rolling element faults up to 0.028” diameter. Among the 500 samples in 10 categories, a total of 496 samples were correctly classified, and the classification accuracy rate reached 99.2%. The categories of faults 1–4 and 7–10 were correctly classified. The samples were correctly classified, while the remaining four were misclassified into fault category 5. Fault 5 and fault 6 are faults of the inner and outer rings at 0.014 inches, respectively, and these two types of faults are easily confused during diagnosis. Figure 6 shows the 2D scatter diagram of the classification of the HPSO-CNN-LSTM model. It can be observed that the features extracted by the HPSO-CNN-LSTM model have a good clustering effect. The clusters are relatively independent in different states and can be clearly distinguished in the ten classification states. The above results prove that the HPSO-CNN-LSTM model has high classification accuracy and can effectively diagnose fault types.

### 4.3. Comparison of Different Deep Learning Network Models

To evaluate the proposed model performance, conventional CNN, LSTM and CNN-LSTM models are used and compared with the HPSO-CNN-LSTM model. The same test set was classified, and their classification accuracies were calculated. Table 2 shows the classification accuracies of the four different models for fault diagnosis. The accuracies of the CNN, LSTM, CNN-LSTM and HPSO-CNN-LSTM models are 92.6%, 90%, 94.2% and 99.2%, respectively. The CNN-LSTM model optimized by HPSO has the highest accuracy rate, 6.6%, 9.2% and 5% higher than the other three models, respectively. Therefore, it can be concluded that the model combining CNN-LSTM shows a better fault diagnosis effect than a single model, and the HPSO-CNN-LSTM model further improves the model by dynamically adjusting the hyperparameters of the CNN-LSTM model. Classification accuracy and fault diagnosis performance.

**Figure 7:**
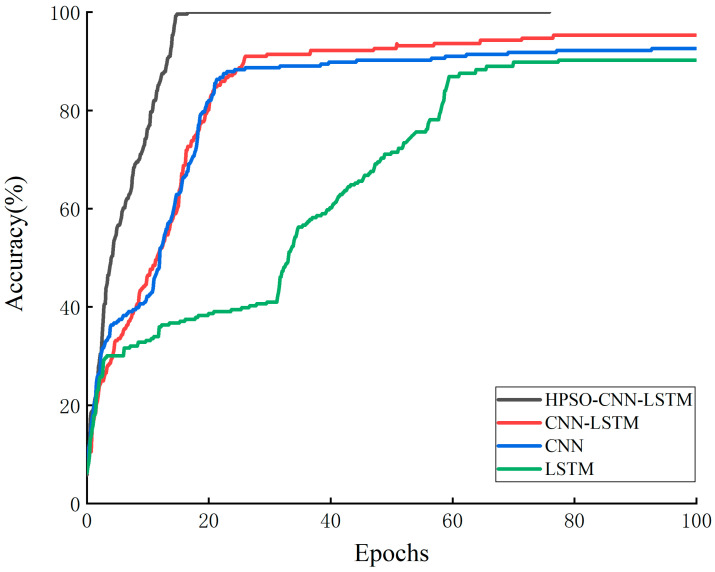
Accuracy curves of training sets of different network models.

Figure 7 shows the accuracy rate curves of the four network models on the training set. It can be seen that the iteration speed of the traditional network model is slow, and the accuracy rate is low. The LSTM model has the slowest iteration speed and requires approximately 100 iterations to reach a 90% accuracy rate. The CNN model can reach a 92.6% accuracy rate after approximately 30 iterations. The CNN-LSTM model is better than the single network models, reaching a 94.2% accuracy rate after approximately 30 iterations, while the HPSO-CNN-LSTM model can achieve an accuracy rate of 99.2% after approximately 15 iterations. Therefore, the HPSO-CNN-LSTM model improves the classification accuracy and speeds up the iteration speed.

**Figure 8:**
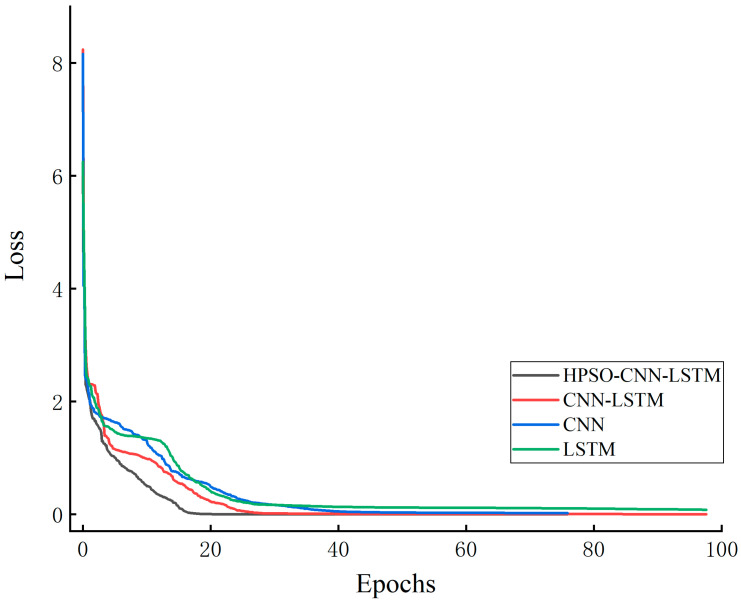
Loss curves of training sets of different network models.

Figure 8 shows the loss function curves of the four different network models on the training set. It can be observed that the CNN-LSTM model has a faster convergence speed than the separate CNN and LSTM models, while the HPSO-CNN-LSTM model shows stronger convergence and the fastest convergence speed. This is because the parameters of the CNN-LSTM model are optimized through HPSO, and each parameter is adjusted to the most suitable value to make it more compatible with the CNN-LSTM model. This model improves the accuracy rate and reduces the error and training speed.

### 4.4. Comparison of Network Models with Different Optimization Parameters

**Figure 9:**
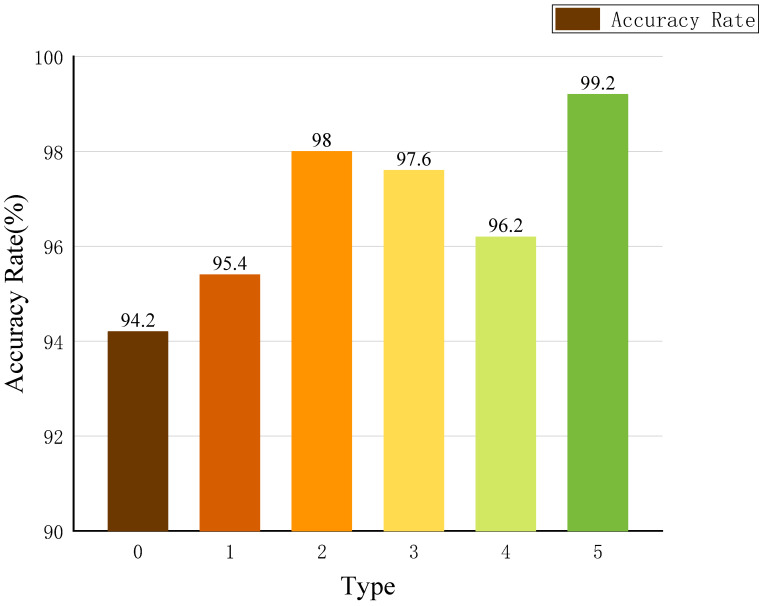
Classification accuracy of the network model with different optimization parameters.

For the optimization of the HPSO-CNN-LSTM model, we divided the four optimization parameters into four categories for testing. Among them, categories 1, 2, 3, 4, and 5 correspond to the number of HPSO-optimized convolution kernels, the number of hidden layer nodes, the learning rate, the maximum number of iterations, and the CNN-LSTM network model that optimizes four parameters at the same time. The initial CNN-LSTM network model corresponds to category 0. The initial parameter settings of the model are shown in Table 1, and the unoptimized parameters are shown in Table 3. Table 4 shows the parameter settings and accuracy of six different network models, and the histogram of accuracy is shown in Figure 9.

We compared the fault diagnosis accuracy of the HPSO-CNN-LSTM model with the four parameters optimized simultaneously and separately with the traditional CNN-LSTM model. According to the results in Table 4, HPSO optimized the four parameters of the CNN-LSTM model. Compared with the traditional CNN-LSTM model, the accuracy rate can be significantly improved by 1.2%, 4.2%, 3.4%, and 2%, respectively. When HPSO is used to optimize the four parameters of CNN-LSTM at the same time, the accuracy rate is the highest, reaching 99.2%. Compared with the traditional CNN-LSTM model and the other four optimization models, the accuracy rate is increased by 5%, 3.8%, and 1.2%, respectively. Figure 9 clearly shows the difference in accuracy between different types of models. The accuracy of the CNN-LSTM model after HPSO optimizes four parameters at the same time is the best. This proves that when HPSO optimizes the four parameters of CNN-LSTM at the same time, the model diagnosis effect is the best. Therefore, the HPSO-CNN-LSTM model proposed in this paper has stronger fault diagnosis capabilities.

The experimental results show that the CNN-LSTM model optimized by HPSO shows better performance than the traditional neural network and the CNN-LSTM model under different parameter settings. The optimized model parameters can be dynamically adjusted during training, making the network model more stable and achieving better classification accuracy. Substituting the parameters obtained after HPSO optimization into the CNN-LSTM model and using this model to diagnose bearing faults, the optimal performance can be achieved. Therefore, the HPSO-CNN-LSTM model proposed in this paper has higher classification accuracy and better fault diagnosis ability.

## 5. Conclusions

This study proposes a rolling bearing fault diagnosis model based on HPSO-CNN-LSTM. The model uses the HPSO algorithm to optimize the parameters of the CNN-LSTM model. Based on the standard particle swarm optimization algorithm, the HPSO algorithm introduces the crossover and mutation operations of the genetic algorithm, which overcomes the problem that the PSO algorithm easily falls into a local optimum and has a strong global optimization ability. Aiming at the problem that the parameters of the CNN-LSTM model are difficult to determine and cannot be adjusted with training. The diagnostic accuracy corresponding to different parameters is also relatively large. The HPSO algorithm is used to optimize the number of convolution kernels, the number of hidden layer nodes, and the number of hidden layer nodes in the CNN-LSTM model. learning rate and a maximum number of iterations and applied the model to a diagnostic study on rolling bearing fault data from Case Western Reserve University. Experimental results show that the HPSO-CNN-LSTM model has high diagnostic performance, and the fault classification accuracy rate reaches 99.2%.

Compared with other deep learning network models, the HPSO-CNN-LSTM model has the following advantages:

First, compared with the GA and PSO algorithms, the HPSO algorithm has stronger global optimization ability, avoids premature convergence problems, and can better solve nonlinear and multivariate optimization problems. Regarding CNN-LSTM model parameter optimization, the HPSO algorithm shows better applicability. The parameters of the CNN-LSTM model are optimized through the HPSO algorithm. The optimal parameter value is dynamically searched, which makes up for the deficiency that the parameters of the CNN-LSTM model cannot be adjusted dynamically, overcomes the uncertainty of manually adjusting the model parameters, and improves the failure of the model diagnostic accuracy and faster iterations.

Second, the experimental results show that the HPSO-CNN-LSTM model has better diagnostic performance, can achieve higher precision and accuracy, and has a faster iteration speed. Compared with the traditional CNN, LSTM and CNN-LSTM models, the fault diagnosis accuracy of this model is increased by approximately 6.6%, 9.2% and 5%, respectively. In addition, by comparing the network models with different optimization parameters, it is found that when the four parameters are optimized at the same time, the HPSO-CNN-LSTM model has the highest accuracy and the best fault diagnosis performance.

In summary, compared with the CNN-LSTM model and other traditional deep learning network models, the HPSO-CNN-LSTM model has a better effect on rolling bearing fault diagnosis and can achieve more accurate fault diagnosis, thereby significantly improving fault diagnosis, efficiency and safety. The limitation of this model is that the process of parameter optimization by the HPSO algorithm is computationally intensive. With enhanced computer capabilities, this method can be extended to more complex network models. In the future, research will continue to accelerate algorithm optimization and improve model performance.

## Figures and Tables

**Table 1:** HPSO-CNN-LSTM network model parameters.

Layer Type	Network Parameters	Optimized Parameters
Input layer	[4096, 1, 1]	Activation = ‘relu’ Classifier = ‘softmax’ Optimizer = ‘AdamOptimizer’
CNN layer	2	
Pooling layer	Maxpooling, pooling length = 2, Stride = 2	
LSTM layer	2	
Output layer	10	
Optimized parameters	Number of filters = 6, 6 Number of hidden layer units = 197, 197	Learning rate = 0.0649 Max epochs = 76

**Table 2:** Accuracy rate in the testing dataset of different models.

Model	CNN	LSTM	CNN-LSTM	HPSO-CNN-LSTM
Accuracy rate (%)	92.6	90	94.2	99.2

**Table 3:** CNN-LSTM initial parameters.

	Network Parameters	Optimize Parameters
CNN-LSTM initial parameters	Number of filters = 8, 16 Number of hidden layer units = 100, 100	Learning rate = 0.1 Max epochs = 100

**Table 4:** Accuracy of the network model with different optimization parameters.

Category	Number of Filters	Number of Hidden Layer Units	Learning Rate	Max Epochs	Accuracy Rate
0	8, 16	100, 100	0.1	100	94.2%
1	6, 6	100, 100	0.1	100	95.4%
2	8, 16	197, 197	0.1	100	98%
3	8, 16	100, 100	0.0649	100	97.6%
4	8, 16	100, 100	0.1	76	96.2%
5	6, 6	197, 197	0.0649	76	99.2%

## Data Availability

The data presented in this study are openly available in the Case Western Reserve University Bearing Data Center at https://engineering.case.edu/bearingdatacenter/download-data-file, accessed on 15 June 2023.
